# Herbal Waste from Filter-Tea Production as Eco-Friendly Ash for Sustainable Natural Rubber Composites

**DOI:** 10.3390/ma18010204

**Published:** 2025-01-06

**Authors:** Jelena Lubura Stošić, Oskar Bera, Teodora Vukša, Dario Balaban, Senka Vidović, Aleksandra Gavarić, Sanja B. Ostojić, Siniša Simić

**Affiliations:** 1 Faculty of Technology Novi Sad, University of Novi Sad, Bulevar Cara Lazara 1, 21000 Novi Sad, Serbia; obera@uns.ac.rs (O.B.); teodora.vuksa@gmail.com (T.V.); dario.balaban@uns.ac.rs (D.B.); senka.vidovic@uns.ac.rs (S.V.); cvejina@uns.ac.rs (A.G.); sinisa.simic@uns.ac.rs (S.S.); 2 Faculty of Technology Zvornik, University of East Sarajevo, Karakaj 34A, 75400 Zvornik, Republic of Srpska, Bosnia and Herzegovina; 3 Institute of General and Physical Chemistry, University of Belgrade, Studentski Trg 12-16, 11000 Belgrade, Serbia; ostojicsanja404@gmail.com

**Keywords:** filter-tea production waste, natural rubber, composites, ash, herbal waste, mechanical properties, green tea, hibiscus, lemon balm

## Abstract

Herbal dust, a waste byproduct from filter-tea production, was annealed to form ash that can be incorporated into natural rubber as an eco-friendly filler. Three types of herbal dust ash (HDA), green tea, hibiscus, and lemon balm, were added at two different contents, 2.5 and 5 phr, into the rubber compound, while the content of carbon black, as a filler, was maintained at 50 phr in all samples. The impact of HDA type and content on the rheological and mechanical properties of rubber products was evaluated. Rheological analysis showed that HDA samples exhibited slightly lower maximum torque values (around 11.6 dNm) than ash-free samples (13.53 dNm), yet maintained vulcanization effectiveness with minimal impact on torque or cure rate metrics. Mechanical testing found that samples with 2.5 phr of lemon balm ash achieved comparable properties to samples without added ash, while samples with added hibiscus preserved crosslinking density and hardness. The addition of HDA led to decreases in tensile strength, elongation at break, and hardness values, with slight changes suggesting its applicability in similar industrial contexts. The findings highlight HDAs potential as a cost-effective, sustainable filler for rubber production, contributing to circular economy practices by repurposing significant amounts of tea waste into high-quality rubber materials.

## 1. Introduction

Tea, as the most widely consumed non-alcoholic beverage in the world, is experiencing rising demand, which increases the need for effective tea waste disposal solutions. With growing tea production, large amounts of waste are generated annually, and disposing of this waste is challenging. The majority of tea waste ends up in landfills, is composted, or is incinerated, resulting in both significant product waste and negative impacts on the environment [1,2]. The production of filter tea generates waste in the form of finely powdered plant material with particle sizes below 0.315 mm [3]. This material, known as herbal dust, is often regarded as waste solely because of its small particle size. The amount of herbal dust produced varies based on the plant material’s type and species, accounting for 10% to 40% of the total processed material [4]. Various efforts have been made to repurpose this material, such as use as an adsorbent for Cu and Pb removal from wastewater [5], as a cost-effective substrate for oyster mushroom cultivation [6], and for hydrogen production [7,8,9]. Since tea waste still contains active components, our previous research [10] focused on extracting valuable components from it. In a previous study, the quality of extracts from the herbal dust of green tea (*Camellia sinensis*), hibiscus (*Hibiscus sabdariffa*), and lemon balm (*Melissa officinalis*) was evaluated. However, extraction leaves behind residual waste that must be addressed. Integrating this post-extraction waste into rubber compounds as ash may offer a solution to the growing tea waste problem while promoting a circular economy [1].

Rubber is a material with diverse applications, mostly used for tires, clothing, and footwear. Its wide range of uses is attributed to key properties like high tensile strength and elasticity, which are essential for many purposes [11]. In order to be effectively used for different causes, natural rubber must go through the vulcanization process, which increases the elasticity of the material and ensures its recovery after deformation. This is made possible by the formation of a three-dimensional molecular network through covalent crosslinking of linear rubber polymer chains [12,13]. During the vulcanization process, fillers are added to rubber compounds to improve their physical properties. Carbon black (CB) is most commonly used as a rubber filler due to its purity, leading to enhanced performance of this material as well as its low cost. Nonetheless, incorporating a cheaper filler could benefit the rubber industry significantly [14,15,16,17]. Various authors [18,19,20] have studied the effects of incorporating herbal waste and ash into rubber mixtures as a partial replacement of CB, aiming to lower production costs and develop a bio-based product. Nevertheless, all findings indicate that every addition of new materials into the rubber compound changes the properties of the final product, mostly affecting its tensile strength, scorch, cure times, and hardness. This results in some limitations on the rubber’s applications after production, as different industries demand specific properties. Carbon black has a high surface area, which directly affects the properties of the rubber product, and has a beneficial role in rubber-filler interactions due to its polar functional groups that provide effective reinforcement. As ash particles are larger in size, therefore having a smaller specific surface area, and show lower rubber-filler interactions, it is clear that herbal ash cannot be a replacement for CB. However, incorporating tea waste ash could significantly reduce rubber production costs, transforming this by-product of filter tea production into a valuable resource for the rubber industry [19,21].

In this work, herbal dust, a byproduct from the ultrasound-assisted extraction examined in a previous study [10], was converted to ash by annealing at 800 °C. The focus was to investigate the impact of different herbal dust ash on rubber properties. Variations in herbal dust ash type (green tea, hibiscus, and lemon balm) and its content were used to assess their impact on the thermal, rheological, and mechanical properties of natural rubber products. A literature review provided examples of incorporating various types of ash into elastomeric matrices to produce natural and synthetic rubber [22,23,24,25,26,27,28]. However, no literature addresses the addition of ash derived from herbal dust from filer-tea production or herbal dust post-extraction into the natural rubber mixture. Based on the results, the effect of herbal ash on the rheological and mechanical properties of the prepared rubber mixtures was observed. The data indicated that small amounts of ash can be incorporated into natural rubber compounds without compromising vulcanization or mechanical properties. As the findings demonstrate that ash derived from herbal dust can be incorporated in rubber mixtures while maintaining comparable end-use characteristics to ash-free composites, these results hold significant environmental importance. This approach supports sustainable rubber production, enhances the circular economy, and transforms waste from the filter-tea industry into a valuable component in rubber composites.

## 2. Materials and Methods

Prepared rubber mixtures consist of natural rubber Standard Vietnamese Rubber CV60 (Vietnam Rubber Group, Ho Chi Minh City, Vietnam) and incorporate the following additives obtained from Edos (Zrenjanin, Serbia), which are used without further purification: zinc oxide (ZnO), stearic acid, N-isopropyl-N’-phenyl-p-phenylenediamine (IPPD), Sulfur, and N-cyclohexylbenzothiazol-2-sulfenamide (CBS). Carbon black N330 was supplied from Nhumo (Altamira, Mexico), and the preparation of herbal dust ash is described in detail in the next section. All raw materials used in the research, except for herbal dust ash, are commercially available and widely used in the rubber industry.

### 2.1. Preparation of the Herbal Dust Ash

Herbal dusts of green tea (*Camellia sinensis*) leaves, hibiscus (*Hibiscus sabdariffa*) calyx, and lemon balm (*Melissa officinalis*) leaves were obtained from a local tea manufacturer, Fructus, Bačka Palanka, Republic of Serbia. In the previous study [10], ultrasound-assisted extraction was utilized for the extraction of green tea, hibiscus, and lemon balm herbal dust from the filter-tea industry. The findings indicated that herbal dust, which was previously considered waste, can be effectively extracted, yielding bioactive compounds at concentrations comparable to those in commercially available plant materials, as described in detail in the study [10]. While this waste material proves useful due to its high bioactive compound content, there remains residual waste post-extraction. Therefore, herbal dust waste after extraction is first exposed to annealing in open air at a temperature of 500–700 °C in order to prevent flame inside a furnace. The average weight loss during annealing in an open air for green tea, hibiscus, and lemon balm was 90.44%, 66.13%, and 82.58%, respectively. Based on the weight loss of herbal dust, it was observed that the majority of the organic matter evaporated. After annealing in open air, herbal dust ash was exposed to annealing in a furnace with circulating air at a temperature of 800 °C, where the average weight loss during annealing in the furnace for green tea, hibiscus, and lemon balm was 12.47%, 6.79%, and 11.58%, respectively.

### 2.2. Composition of the Natural Rubber Mixtures

In order to investigate the potential use of eco-friendly herbal dust ash, it was incorporated into a rubber compound with varying content. In the rubber industry, the relative composition of the mixture is expressed by assuming that the basic ingredient of the mixture is the rubber and is denoted by 100, while the amount of other components is represented in relation to the amount of rubber, and the thus obtained unit is denoted by phr (parts per hundred rubber). The environmentally friendly herbal dust ashes (green tea, hibiscus, and lemon balm) were varied to 2.5 and 5 phr, while the amounts of other components remained constant. The prepared natural rubber samples were labeled according to herbal dust ash content, as indicated in Table 1.

The recipe for obtaining natural rubber mixture samples is presented in Table 2. The component masses shown in Table 2 are calculated according to the recipe in phr units (second column of Table 2), with a filling factor of 0.75 for the mixing chamber to ensure stability during the mixing process. The filling factor represents the ratio of the mixture’s volume to the empty chamber’s volume. In Table 2, the components are categorized into inactive and active groups, where carbon black, herbal dust ash, ZnO, stearin, and IPPD are considered inactive, while sulfur and CBS are categorized as active components [29].

### 2.3. Mixing Procedure

The mixing procedure involves several steps, during which the mixer temperature is maintained at 90 °C to prevent the initiation of vulcanization. The first step represents mixer preparation, idle run, with rotation rotor speed of 30 min^−1^. Upon completion of the first mixing step, the natural rubber, measured according to Table 2, is added to the mixing chamber; the rotor speed was increased to 100 min^−1^ and the natural rubber was mixed for three minutes; then the speed was reduced to 60 min^−1^ and mixed for another three minutes. During the second and third stages, when only the rubber is mixed, its mastication occurs, which enables a better dispersion of the other components of the mixture during the following mixing steps. In the fourth step, with the rotor speed remaining constant, the inactive components of the rubber mixture are added and mixed for five minutes. In the final step, the active components are added and mixed for two minutes while maintaining the same rotor speed. At the end of the process, the chamber is opened, and the device is turned on again at 30 min^−1^ to facilitate the extraction of the mixture. The specified mixing procedure was described in detail in [30] and enables optimal dispersion of all components and even distribution of filler in the polymer matrix. The resulting mixture is cooled and stored at room temperature for 24 h to ensure conditioning before vulcanization is performed [31].

### 2.4. Vulcanization Procedure

The natural rubber nanocomposite samples were vulcanized according to ISO 37 standard [32]. The process involved pressing the rubber sheets at 150 °C and atmospheric pressure for 15 min. Following this, the vulcanized sheets, each with a thickness of 2.2 mm, were left at room temperature for 24 h to allow any additional crosslinking reactions to complete before being cut into dumbbell shapes, according to ISO 37.

### 2.5. Characterization

The elementary composition and morphology of the samples, three different herbal dust ashes (green tea, hibiscus, and lemon balm), and prepared vulcanized natural rubber samples were investigated by SEM using a JEOL JSM-6460LV at 25 kV, combined with a microanalysis method using X-ray energy dispersion (EDS). The samples were prepared for analysis by slicing them into sections, coating them with a thin layer of gold using the SCD-005 coating system (Bal-tec/Leica, Wetzlar, Germany), and examining them under varying magnifications.

### 2.6. Rheological Measurements

The vulcanization process of all prepared samples was monitored during 15 min at 150 °C using an oscillating MDR-A Rotorless Rheometer, supplied by Beijing Rade Instrument Co., Ltd., Beijing, China.

### 2.7. Mechanical Properties Measurements

To determine the mechanical properties of the composites with varying herbal dust ash content, tensile strength, elongation at break, and hardness were tested.

Tensile strength and elongation at break were measured according to ISO 37 using the dynamic Rade extensometer, RT5K-2, manufactured by Beijing Rade Instrument Co., Ltd., Beijing, China. Each sample was tested five times, and the average value was calculated.

The hardness of prepared vulcanized samples with different herbal dust ash content was tested following the ISO 7619-1 standard [33] using a Shore A durometer, model Zwick 3100, manufactured by Zwick, Ulm, Germany. The hardness measurements were conducted three times to obtain an average value, which is expressed in Shore A units.

### 2.8. Swelling Tests

The swelling behavior of prepared natural rubber nanocomposite was determined by the change in mass using the following equilibrium method. Vulcanized samples with an approximate weight between 190 and 220 mg were immersed in 40 mL of pure toluene during the 72 h observation period at room temperature. Samples were removed from the toluene bath after 72 h, quickly dried to remove toluene from the surface, and weighted in order to obtain data for the calculation of the swelling index [34]:

(1) $$SI,\;\%=\frac{w_{s}-w_{0}}{w_{0}}\times 100\%$$

where *SI* is the swelling index (%), *w_s_* is the mass of the swollen sample (grams) after 72 h swelling time, and *w*_0_ is the initial mass of the sample (grams).

After measuring sample weight after swelling, samples were put in an over with circulating air at 70 °C in order to remove absorbed toluene. After the toluene was removed from the samples, sample weight was measured in order to calculate crosslinking density, which represents the molar number of elastically effective network chains per cubic centimeter. Crosslinking density was calculated using the Flory-Rehner equation [35] for the equilibrium state of swollen vulcanized samples, as expressed in the following relation:

(2) $$v_{e}=\frac{-\left[\ln\left(1-V_{r}\right)+V_{r}+\chi V_{r}^{2}\right]}{\frac{V_{s}(V_{r}^{1/3}-V_{r})}{2}}$$

where $v_{e}$ represents crosslinking density (mol/cm^3^), χ is the polymer-toluene interaction parameter, which is taken as 0.38 in this research [36], $V_{s}$ is molecular volume of the sample (106.2 cm^3^/mol), and $V_{r}$ is the volume fraction of a natural rubber in a swollen network in equilibrium with pure solvents and can be calculated as follows:

(3) $$V_{r}=\frac{\frac{w_{dr}}{\rho_{dr}}}{\frac{w_{dr}}{\rho_{dr}}+\frac{w_{at}}{\rho_{t}}}$$

where $w_{dr}$ and $\rho_{dr}$ are weight (grams) and density (0.9125 g/cm^3^) of dry rubber samples, respectively, $w_{at}$ is weight of solvent absorbed by sample (grams), calculated as the difference in weight after swelling, before and after drying, and $\rho_{t}$ is toluene density (0.867 g/cm^3^).

Shear modulus (MPa) can be calculated as follows:

(4) $$G=\frac{RT\rho_{dr}}{M_{p}}$$

where *R* is the universal gas constant (8.314 J/molK), *T* is the absolute temperature (273 K), and *M_p_* is the average molecular weight of the polymer between crosslinks and can be calculated using the following equation:

(5) $$M_{p}=\frac{\rho_{dr}}{v_{e}}$$

### 2.9. Thermal Analysis

Differential scanning calorimetry (DSC) measurements were performed on the DSC Q 1000 differential scanning calorimeter with the RCS cooling system from TA Instruments, New Castle, DE, USA. The instrument was calibrated for temperature and enthalpy according to the standard instructions of the manufacturer using standard metal indium, whose melting point temperature is T = 156.59 °C and enthalpy of melting ΔH = 28.18 J/g [37]. All experiments were conducted in the nitrogen flow (purity 99.999%) at a gas flow rate of 50 mL/min in a DSC cell. Samples for DSC analysis were cut and weighed to the mass of 10–13 mg by the analytical scale balance Mettler Toledo AE 163, Toledo. The samples were equilibrated at −90.00 °C, then heated to 150 °C with a heating rate of 10 °C/min, equilibrated for 1 min, and then cooled to −90 °C. After equilibration at −90 °C for 1 min, the second heating scan to 150 °C with a heating rate of 10 °C/min was performed. Each thermogram was analyzed using TA Advantage Universal Analysis 2000 software in order to obtain the samples’ glass transition (*T_g_*) temperatures: onset temperature (*T_g, on_*), midpoint temperature (*T_g_*), and glass transition final temperature (*T_g, end_*).

## 3. Results and Discussion

### 3.1. The Morphology of Herbal Dust Ashes and Natural Rubber Composites

**Figure 1:**
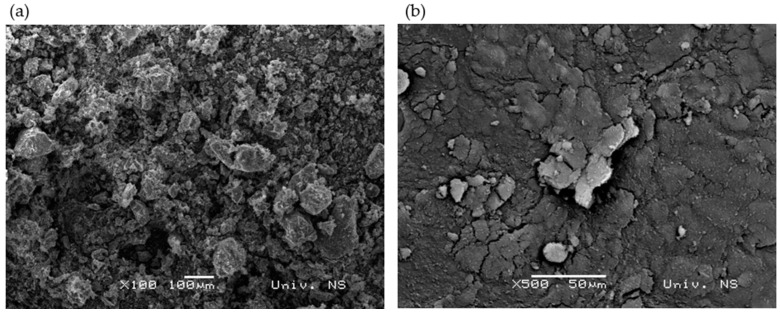
SEM image of (**a**) hibiscus ash and (**b**) carbon black.

Filter-tea production generates waste in the form of powdered plant material, which is called herbal dust, and it is often considered a waste only due to its particle size. Within a previous study [10], ultrasound-assisted extraction was applied for the extraction of green tea, hibiscus, and lemon balm herbal dust from the filter tea industry. In the current research, the post-extraction waste was used and transformed into ash by two-step annealing. The surface morphology of hibiscus dust ash is presented in Figure 1a, and since the morphology of green tea and lemon balm showed no difference from hibiscus ash, it is presented in Figure S1 in the Supplementary Material.

**Figure 2:**
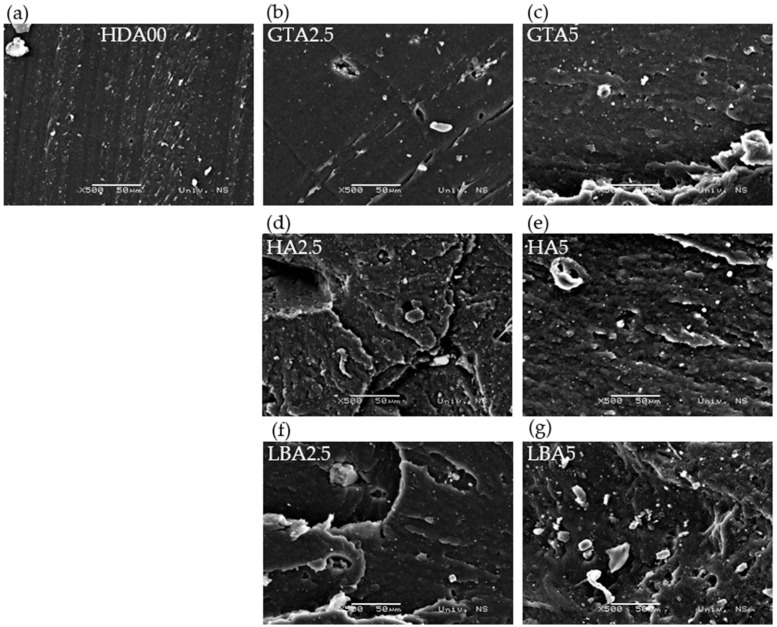
SEM images of natural rubber samples: (**a**) HDA00, (**b**) GTA2.5, (**c**) GTA5, (**d**) HA2.5, (**e**) HA5, (**f**) LBA2.5, (**g**) LBA5.

The morphology analysis of the herbal dust showed no difference in particle size between green tea, hibiscus, and lemon balm, and EDS analysis indicated no variation in composition, as only carbon was detected. There may be trace amounts of nitrogen and oxygen in the composition of the herbal ash; however, these quantities are so small that they remain undetectable. This result was expected, given that the material was annealed in a furnace at 800 °C. Figure 1 highlights the differences between hibiscus ash and carbon black particles. At 100× magnification, hibiscus ash particles are visible and appear agglomerated, while carbon black particles are in fine powdery form, with sizes ranging from 30 to 60 nm. Observing the carbon black particles requires higher magnification, as illustrated in Figure S2 of the Supplementary Material. The samples were prepared according to the recipe shown in Table 2 and vulcanized at 150 °C for 15 min. They were then cooled for 24 h to ensure the completion of all crosslinking reactions, which persist until the entire rubber product has cooled below the vulcanization temperature [31]. After the vulcanization process, the morphology of all prepared samples was recorded using SEM analysis, as shown in Figure 2. The scanning electron microscope utilizes a concentrated electron beam to examine the surface of composite materials. When the electrons interact with the sample, they produce signals that enable the generation of detailed, high-resolution images. This technique is particularly valuable for analyzing filler dispersion, as it can reveal particle aggregation and size distribution within composites [38].

Figure 2a shows that matrix constituents are dispersed throughout the natural rubber. In contrast, the presence of large microporous herbal ash particles disrupts the continuous rubber phase, leading to noticeable discontinuities in the polymer matrix. The similar continuous phase is observed when 2.5 phr of green tea dust ash is added (Figure 2b), while its further addition is causing larger agglomerates (Figure 2c). The addition of hibiscus ash is disrupting the natural rubber continuous phase, as can be observed in Figure 2d,e. The addition of lemon balm dust ash is further disrupting the rubber matrix phase, which can lead to changes in mechanical properties. As shown in Figure 2, carbon black exhibits greater compatibility with the rubber matrix compared to the studied types of herbal dust ashes. Therefore, it is essential to add the ash in small amounts to ensure optimal performance. The slight variations in the composition of different dust types could lead to disruption of the rubber matrix, impacting both rheological and mechanical properties. Microanalysis using X-ray energy dispersion revealed only carbon as the dominant element after annealing, with trace amounts of other elements likely present but undetectable by this method. This assumption of compositional differences is supported by the visibly distinct colors of the ash residues, as shown in Figure S3 of the Supplementary Material. The different ash colors likely result from undetected compositional variations. Additionally, it can be observed that when comparing herbal dust, green tea ash has a more uniform structure compared to other herbal ashes incorporated into the rubber matrix. The presented morphology should be interpreted with caution, as it is magnified 500 times and represents only a small portion of the sample, not the entire sample.

### 3.2. Rheological Properties of Incorporated Herbal Dust Ash Nanocomposites

**Figure 3:**
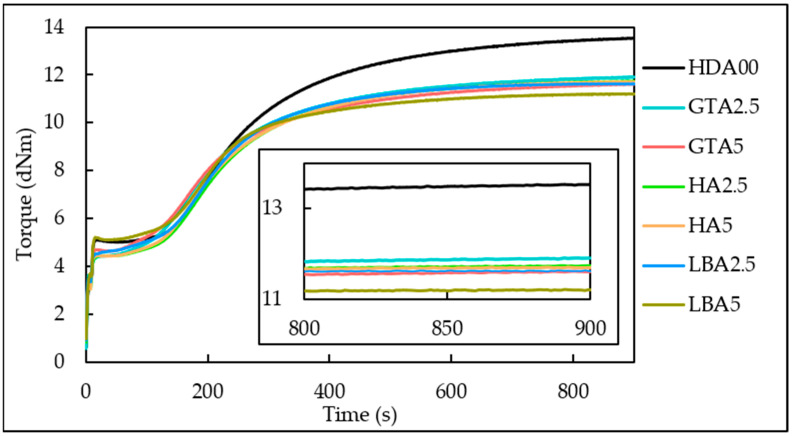
Vulcanization curves, obtained during 15 min at 150 °C.

The impact of incorporating herbal dust ash on the viscoelastic and curing properties was assessed through vulcanization tests conducted using a moving die rheometer at 150 °C during 15 min. These parameters were chosen since they represent the standard procedure employed in this research to produce vulcanized samples. Figure 3 presents a comparison of the rheological torque variations over time for each of the tested natural rubber samples. As shown in Figure 3, every sample exhibits an increase in rheological torque over time, a typical behavior of the vulcanization process. The vulcanization curves, whether with or without added HDA, exhibit a steep and S-shaped profile. The sample without added herbal dust ash demonstrates notably higher overall and final torque values. The HA5 sample, containing 5 phr of hibiscus ash, exhibited a slightly slower vulcanization compared to the other samples filled with HDA. This can be attributed to the slightly different composition in nitrogen and oxygen of hibiscus ash if detected, which can cause reduced reinforcing effectiveness [39,40]. The vulcanization curves for the other samples with herbal dust ash were similar but showed slightly lower overall torque compared to the sample without HDA. This indicates that these samples have considerable potential based on their vulcanization behavior.

Since herbal dust ash primarily consists of carbon, with all organic matter evaporating during annealing, EDS analysis detected only carbon. This suggests that different types of ash exhibit similar vulcanization kinetics. However, their behavior differs from samples without added HDA, as carbon black, with its larger particle size and pure carbon composition, exhibits distinct characteristics.

Table 3 summarizes the rheological parameters measured during the vulcanization process of the prepared natural rubber composites. Adding varying amounts of different herbal dust ash in samples results in an increase in minimum torque and a decrease in maximum torque. However, there is no distinct trend in the changes of minimum and maximum torque based on the amount of ash added (as seen in Table 3). The maximum torque remains quite consistent for all samples, regardless of the type or amount of herbal dust ash used. However, it is lower compared to the vulcanized sample without any added HDA.

The degree of chemical crosslinking of vulcanized samples (Δ*M*) can be described as the relative difference between the maximum and minimum torque (Table 3). The sample containing 5 phr of lemon balm ash showed the lowest degree of chemical crosslinking, while the highest Δ*M* was observed for the sample without HDA. The highest Δ*M* values for samples containing HDA were detected in those with 2.5 and 5 phr of added hibiscus ash, although these increases are minimal and cannot be attributed to the ash addition. The pattern of change for Δ*M* does not correlate with the amount of ash added. It is assumed that the quantity of ash incorporated into the samples in this study is too small to detect any trends related to the herbal dust ash addition. A higher content of herbal dust ash was not included in the rubber mixing recipe, as a literature review indicated that a higher ash content would significantly deteriorate the properties of natural rubber [41,42].

Cure rate index (CRI) values, as shown in Table 3, increased with the addition of herbal dust ash. The highest CRI values were observed for the sample containing 5 phr lemon balm, aligning with the maximum torque for this sample.

Scorch time (*t_s_*_2_) is the period required for the initial formation of a wide three-dimensional structure, which imparts elasticity to the material. This metric is crucial in industrial settings, as once scorching occurs, the material loses its plasticity and cannot be reshaped. Consequently, molding and mechanical processing of the rubber mixture must be finalized before the scorch time; otherwise, the material becomes unusable. The GTA5 and LBA5 samples exhibited shorter scorch times, indicating they vulcanized faster compared to other samples (Table 3). If selected for use in the rubber industry, the vulcanization time and temperature should be carefully controlled to prevent scorching before vulcanization.

Optimal curing time (*t*_90_) is the time needed for 90% of crosslinking to occur [43]. Table 3 shows that increasing the herbal dust ash content results in lower *t*_90_ values. These values are very similar for samples containing green tea and hibiscus HDA, while lower *t*_90_ values are observed for the sample with incorporated lemon balm ash.

### 3.3. Mechanical Properties

It has been found that adding prepared herbal dust ash to rubber composite influences vulcanization kinetics by reducing the torque over time, signifying that part of crosslinking reactions are obstructed. To explore the potential of incorporating HDA into rubber products, it is essential to study its impact on mechanical properties, as these characteristics ultimately define the rubber’s intended applications. In practice, the process typically works in reverse, i.e., the rubber properties are customized to suit the final product-specific requirements. The rubber industry formulates a blend based on the client’s specifications, adjusting the type and amount of additives within acceptable standards to achieve optimal properties for the intended application. The desired characteristics of the rubber will vary depending on the unique needs and preferences of each customer. Given the nearly limitless range of possible uses for rubber, where each requires an optimal combination of properties, the concept of incorporating HDA into rubber production holds significant promise. Accurately characterizing its effects will help identify suitable applications for the resulting product. Furthermore, understanding HDAs influence on this particular formulation can help predict its impact on similar rubber blends.

The tested reference sample (HDA00) is without herbal dust ash and represents a commonly used type of rubber for various applications. The mechanical properties measured for this sample served as a baseline to assess how the addition of herbal dust ash affects the rubber’s mechanical characteristics.

The influence of type and content of herbal dust ash incorporated into the natural rubber mixture on mechanical properties, including tensile strength, elongation at break, and hardness, is presented in Table 4.

The addition of herbal dust ash reduces tensile strength; however, observing the sample with hibiscus ash, there is almost no change in tensile strength, even at higher hibiscus ash content. The sample containing 5 phr of lemon balm shows significantly lower tensile strength (19.13 MPa), which may suggest different end-use applications compared to the ash-free sample. On the other hand, adding 2.5 phr of lemon balm results in a tensile strength most comparable to that of the sample without added herbal dust ash.

No specific pattern was observed regarding the addition of herbal dust ash and its effect on elongation at break and hardness. The sample containing 2.5 phr of lemon balm exhibited elongation at break values closest to those of the ash-free sample. Overall, the mechanical properties of the sample with 2.5 phr of lemon balm were the most similar to the sample without added herbal dust ash, indicating potential for similar product applications while supporting a circular economy and contributing to a more sustainable, “green” product.

The variations in mechanical properties among samples containing different types and amounts of herbal dust ash can be attributed to differences in composition. Although primarily composed of carbon, small quantities of other elements present in the ash may influence the mechanical properties by interfering with crosslinking reactions during the vulcanization process.

### 3.4. Swelling of the Herbal Dust Ash Nanocomposites

**Figure 4:**
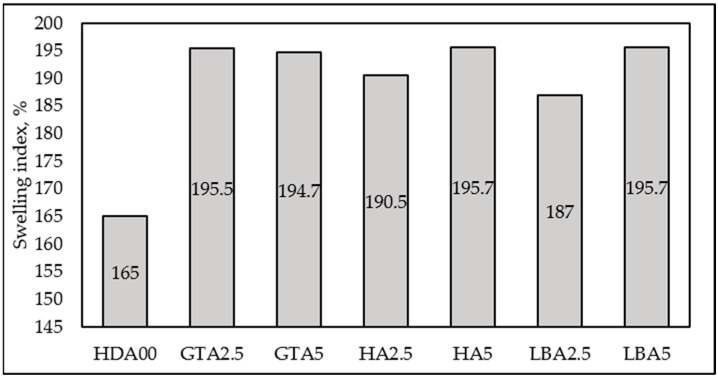
Effect of HDA type and content on Swelling Index.

A swelling test was conducted to observe the interaction between the ash-rubber matrix and to evaluate the crosslinking in vulcanized samples with varying types and amounts of herbal dust ash. The swelling experiments were carried out over a 72 h period, during which the samples were immersed in a toluene bath at room temperature. After the swelling period, the samples were quickly dried to remove toluene from the surface and then weighed to calculate the Swelling Index according to Equation (1). The degree of crosslinking can be directly assessed by the swelling index; a lower swelling index indicates a higher degree of crosslinking. This index represents the amount of solvent absorbed per unit weight of rubber. Figure 4 illustrates the impact of herbal dust ash loading on the swelling index. The results indicate that the penetration of toluene into ash-filled natural rubber samples was increased with the addition of HDA. However, no consistent trend was observed regarding the influence of the type and content of HDA on the swelling index. The sample without added ash exhibited the lowest swelling index, which can be attributed to the superior dispersion of smaller nanosized carbon black particles in natural rubber, enhancing the filler-rubber matrix interaction in the composites [36].

**Figure 5:**
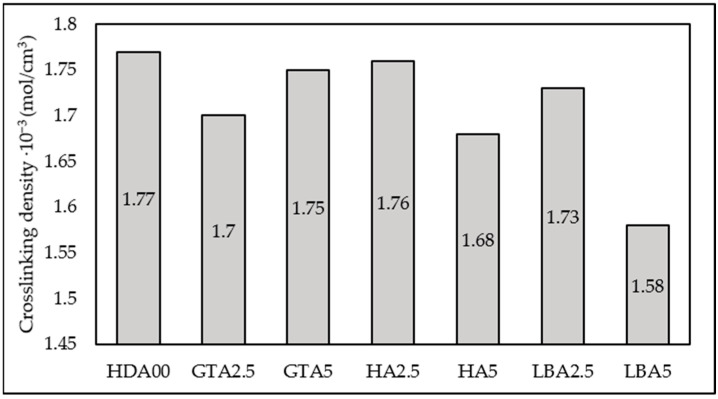
Effect of HDA type and content on crosslinking density.

Determining the crosslinking density from swelling behavior is crucial for understanding the structural properties of a crosslinked polymer. This parameter, which represents the average molecular weight between crosslinks, is directly related to crosslinking density and can be assessed through swelling measurements using Equation (2). The crosslinking density refers to the count of elastically active network chains fully integrated within an ideal network per unit volume, as it is presented in Figure 5. It can be seen from Figure 5 that as the content of the herbal dust ash increases, the crosslinking density decreases, with the exception of the sample containing green tea ash. Previously stated is related to the mobility of the rubber molecules, which is reduced due to added HDA, making it more challenging for toluene to permeate the rubber matrix. The larger particle size of herbal dust ash, in contrast to carbon black, prevents the formation of crosslinks between the natural rubber polymer chains, causing lower values of crosslinking density. The sample containing 2.5 phr of hibiscus ash exhibits a crosslinking density comparable to the sample without added HDA, aligning with their similarly high hardness. Furthermore, the sample containing 2.5 phr of lemon balm ash demonstrates mechanical properties and crosslinking density similar to the sample without HDA. These samples (HA2.5 and LBA2.5) show potential for industrial rubber production, as they result in only minor changes to the rubber material. Additionally, the GTA2.5 and GTA5 samples exhibited the most uniform morphological structure, as shown in Figure 2, which can be related to crosslinking density. This suggests that the green tea particles did not prevent the vulcanization process from occurring. The vulcanization curve (Figure 3) further supports this, as it shows a vulcanization curve that closely resembles that of the ash-free sample and exhibits higher maximum torque (GTA2.5) compared to other samples.

**Figure 6:**
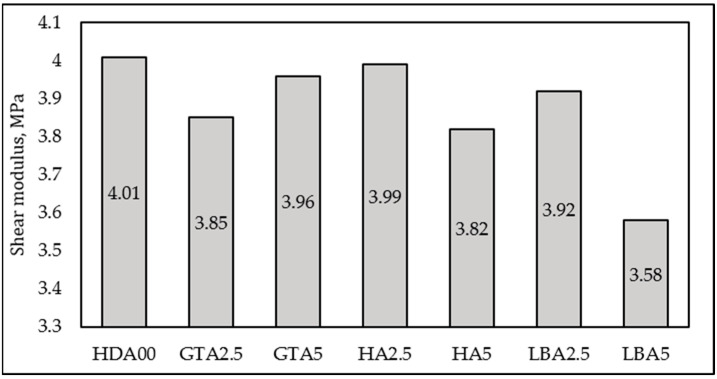
Effect of HDA type and content on shear modulus.

The shear modulus (*G*) of the vulcanized samples was determined through the swelling test using Equation (4), and the resulting values follow the same trend as the crosslinking density, as shown in Figure 6.

### 3.5. Thermal Analysis of Herbal Dust Ash Nanocomposites

**Figure 7:**
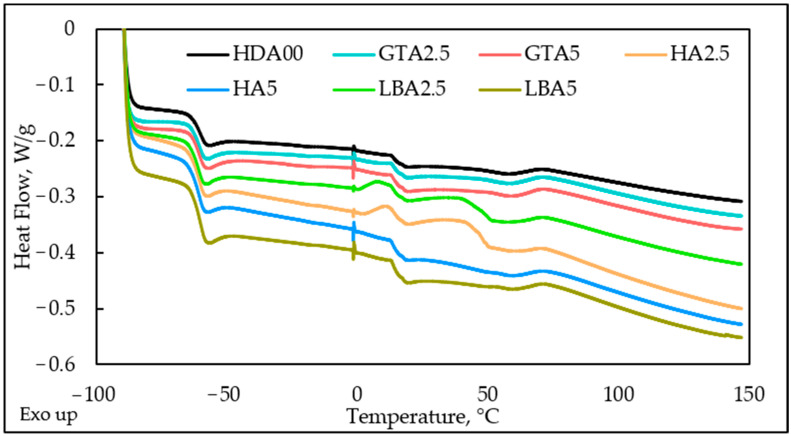
DSC curves of prepared samples with different content and type of herbal dust ash.

To assess the effect of modified physical properties in samples with varying types and amounts of herbal dust ash, glass transition temperature (*T_g_*) is often obtained by DSC technique, as glass transition indicates the onset temperature of rubber chain mobility [44,45,46]. Figure 7 presents the DSC thermogram of vulcanized samples filled with different content and types of herbal dust ash. Table 5 further presents glass transition temperatures, including onset temperature, midpoint temperature, and glass transition final temperature. There are two glass transitions found for all samples on the DCS curves. The first glass transition occurs in the low-temperature region, around −60 °C, which aligns with the findings reported in the literature [46]. The second glass transition, observed at approximately 13 °C, is detected in all investigated samples, including the one without added HDA. This indicates that this *T_g_* is not a result of the HDA addition but rather a characteristic of the natural rubber mixture. This additive disrupts the continuous phase within the rubber matrix, as the larger particle size of the ash, compared to carbon black, prevents the formation of crosslinks between natural rubber polymer chains. As a consequence, material showed phase separation, probably driven by the formation of phase-separated micro-domains, which allowed just a partial miscibility of herbal dust at the molecular level. A slightly lower *T_g_* can be observed for the sample without added herbal dust ash, while this difference is not significant compared to the *T_g_* values of the other samples. The results indicate that dust ash has no significant impact on chain dynamics. 

Interest in renewable resources, especially bioresources, is rapidly growing, particularly for producing sustainable materials, with elastomer science and technology also adapting to this trend. Current literature shows an encouraging shift toward using various natural materials as additives. Additionally, significant potential exists to bridge fundamental research with practical manufacturing applications [47]. The drive for sustainable solutions is prompting industries to re-evaluate their production methods. Consequently, elastomeric compounds based on renewable materials are being increasingly adopted to create environmentally friendly products [48,49]. Traditional fillers in elastomer compositions, used for reinforcement, tend to be quite costly. This makes it appealing to replace them, partially or fully, with fillers from renewable sources, provided that material properties remain intact [50]. If maintaining the natural rubber product’s properties is essential, this research shows that green tea ash provides comparable rheology and crosslinking behavior to samples without added ash. Meanwhile, a sample with 2.5 phr of lemon balm offers similar mechanical properties to an ash-free sample. It should be noted that different properties may be required depending on specific application needs, which could make it feasible to use herbal dust ashes with varying content as long as they meet buyer specifications. Since herbal dust ash is considered waste in the filter-tea industry yet still contains bioactive compounds, it was extracted in research [10], and the post-extraction waste was annealed at 800 °C to prepare ash for incorporation into rubber samples. While traditionally treated as waste, herbal dust shows significant promise in the rubber industry, potentially becoming a valuable component and supporting a circular economy.

## 4. Conclusions

Incorporating herbal dust ash into rubber offers an innovative environmental approach to addressing waste issues from the tea filter industry. By examining the production process and analyzing the rubber’s properties with varying amounts of three types of herbal dust, green tea, hibiscus, and lemon balm, several important conclusions were drawn.

The research findings reveal that adding herbal dust ash disrupts the continuous phase within the rubber matrix, as the larger particle size of the ash, in contrast to carbon black, prevents crosslink formation between natural rubber polymer chains. This distinctive structural impact highlights the potential of herbal ash to produce sustainable rubber materials with adjusted properties.

From a rheological perspective, herbal dust ash influences the vulcanization process by slightly lowering torque values compared to samples without the ash. Nevertheless, similar vulcanization curves among the samples indicate that herbal ash remains supportive of effective rubber processing, retaining essential rheological characteristics desirable for industrial applications.

Mechanical testing further emphasizes the promise of herbal ash as a green additive. For instance, the added 2.5 phr of lemon balm ash closely resembles the mechanical properties of rubber without added ash, suggesting it could be applied in similar product contexts while promoting a circular economy. Additionally, samples with hibiscus and lemon balm ash exhibit crosslinking density and hardness comparable to ash-free rubber, confirming their suitability for industrial use. Furthermore, samples containing green tea ash (GTA2.5 and GTA5) contribute to a more uniform rubber structure without obstructing the vulcanization process, as evidenced by vulcanization curves that closely resemble those of ash-free samples.

The thermal analysis of herbal dust ash nanocomposites showed only slight differences between the samples, which were not significant. This may be due to the small amount of herbal dust ash added; higher concentrations would likely have a greater impact on the rubber matrix.

The incorporation of herbal dust ash, green tea, hibiscus, and lemon balm represents an advancement in sustainable materials science. Not only does it provide an eco-friendly solution for filter-tea waste management, but it also preserves essential natural rubber properties, marking an important step towards the development of green rubber products.

## Figures and Tables

**Table 1:** Labelling of the prepared natural rubber samples.

Sample Code	Herbal Dust Ash	Content, phr
HDA00 *	- *	0
GTA2.5	Green tea ash	2.5
GTA5	Green tea ash	5
HA2.5	Hibiscus ash	2.5
HA5	Hibiscus ash	5
LBA2.5	Lemon balm ash	2.5
LBA5	Lemon balm ash	5

* Sample HDA00, which contains no herbal dust ash, was used as the control sample.

**Table 2:** Recipe for natural rubber mixing.

		HDA00	GTA2.5	GTA5	HA2.5	HA5	LBA2.5	LBA5
Components	phr	Grams						
NR	100	47.91	47.44	46.98	47.44	46.98	47.44	46.98
CB	50	23.95	23.72	23.49	23.72	23.49	23.72	23.49
**HDA**	0, 2.5, 5 *	**0.00**	**1.19**	**2.35**	**1.19**	**2.35**	**1.19**	**2.35**
ZnO	4	1.92	1.90	1.88	1.90	1.88	1.90	1.88
Stearin	1	0.48	0.47	0.47	0.47	0.47	0.47	0.47
IPPD	1	0.48	0.47	0.47	0.47	0.47	0.47	0.47
Sulphur	2.5	1.20	1.19	1.17	1.19	1.17	1.19	1.17
CBS	0.5	0.24	0.24	0.23	0.24	0.23	0.24	0.23

* Herbal dust ash was varied according to Table 1.

**Table 3:** Rheological data of the studied natural rubber samples.

Sample	*M_min_* (dNm)	*M_max_* (dNm)	*ΔM*	*CRI* (min^−1^)	*t_s_*_2_ (min)	*t*_90_ (min)
HDA00	2.87	13.53	10.66	12.80	1.67	8.00
GTA2.5	3.20	11.89	8.69	14.25	1.67	7.25
GTA5	3.28	11.60	8.32	14.34	1.37	7.17
HA2.5	2.97	11.73	8.76	14.15	1.7	7.25
HA5	3.06	11.70	8.64	14.56	1.7	7.05
LBA2.5	3.35	11.63	8.28	15.27	1.85	6.77
LBA5	3.52	11.21	7.69	16.22	1.35	6.35

*M_min_* and *M_max_* are minimum and maximum torque, respectively; Δ*M* is the degree of chemical crosslinking, *CRI* is the cure rate index, *t_s_*_2_ is the scorch time*,* and *t*_90_ is the optimal cure time.

**Table 4:** Mechanical properties of samples with different contents of herbal dust ash.

		HDA00	GTA2.5	GTA5	HA2.5	HA5	LBA2.5	LBA5
Tensile strength (MPa)	Value	27.00	24.06	23.28	24.49	24.54	26.17	19.13
	σ *	0.54	0.93	0.29	1.15	0.70	0.70	0.24
Elongation at break (%)	Value	317.46	277.63	315.03	347.78	317.30	316.02	274.26
	σ *	9.42	3.19	11.79	32.29	14.85	8.93	24.33
Hardness (Shore A)	Value	61.67	60.33	60.33	61.33	59.33	58	58
	σ *	0.58	0.58	0.58	0.58	0.58	1	0

* Standard deviation.

**Table 5:** Glass transition temperatures of samples filled with different content of herbal dust ash.

Sample	*T_g_*_1*, on*_ (°C)	*T_g_*_1_ (°C)	*T_g_*_2*, end*_ (°C)	*T_g_*_2*, on*_ (°C)	*T_g_*_2_ (°C)	*T_g_*_2*, end*_ (°C)
HDA00	−63.09	−59.96	−58.45	13.14	13.65	16.78
GTA2.5	−63.46	−60.27	−59.27	13.15	13.83	16.61
GTA5	−63.32	−60.19	−58.61	13.09	13.78	16.98
HA2.5	−63.84	−60.39	−60.39	12.94	13.47	16.54
HA5	−64.00	−60.68	−59.36	12.83	13.54	16.36
LBA2.5	−63.89	−60.70	−59.63	12.99	13.75	16.91
LBA5	−63.4	−60.14	−58.36	13.06	13.81	16.76

## Data Availability

The original contributions presented in this study are included in the article/supplementary material. Further inquiries can be directed to the corresponding author.

## Supplementary Materials

The following supporting information can be downloaded at: https://www.mdpi.com/article/10.3390/ma18010204/s1, Figure S1: SEM images at different magnification of (a,b) green tea, (c,d) hibiscus, (e,f) lemon balm dust ash; Figure S2: SEM images of carbon black; Figure S3: Herbal dust ash after two-step annealing: (a) green tea, (b) hibiscus, (c) lemon balm.
