# Supplementary material for: Herbal Waste from Filter-Tea Production as Eco-Friendly Ash for Sustainable Natural Rubber Composites
Source: Materials (Basel). 2025 Jan 6;18(1):204. doi: 10.3390/ma18010204 (PMC11722545; doi:10.3390/ma18010204)
Supplement: Supplementary file 1 [file materials-18-00204-s001.zip › materials-3396706-supplementary.pdf]

SUPPLEMENTARY INFORMATION

Herbal Waste from Filter-Tea Production as Eco-Friendly Ash for Sustainable Natural Rubber Composites

Jelena Lubura Stošić [1*], Oskar Bera [1], Teodora Vukša [1], Dario Balaban [1,2], Senka Vidović [1], Aleksandra Gavarić [1], Sanja B. Ostojić [3], Siniša Simić[1]

[1] University of Novi Sad, Faculty of Technology Novi Sad, Bulevar cara Lazara 1, 21000 Novi Sad, Serbia
[2] University of East Sarajevo, Faculty of Technology Zvornik, Karakaj 34A, 75400 Zvornik, Republic of Srpska, Bosnia and Herzegovina
[3] University of Belgrade, Institute of General and Physical Chemistry, Studentski Trg 12-16, RS-11000, Belgrade, Serbia
* Correspondence: jelenalubura@uns.ac.rs

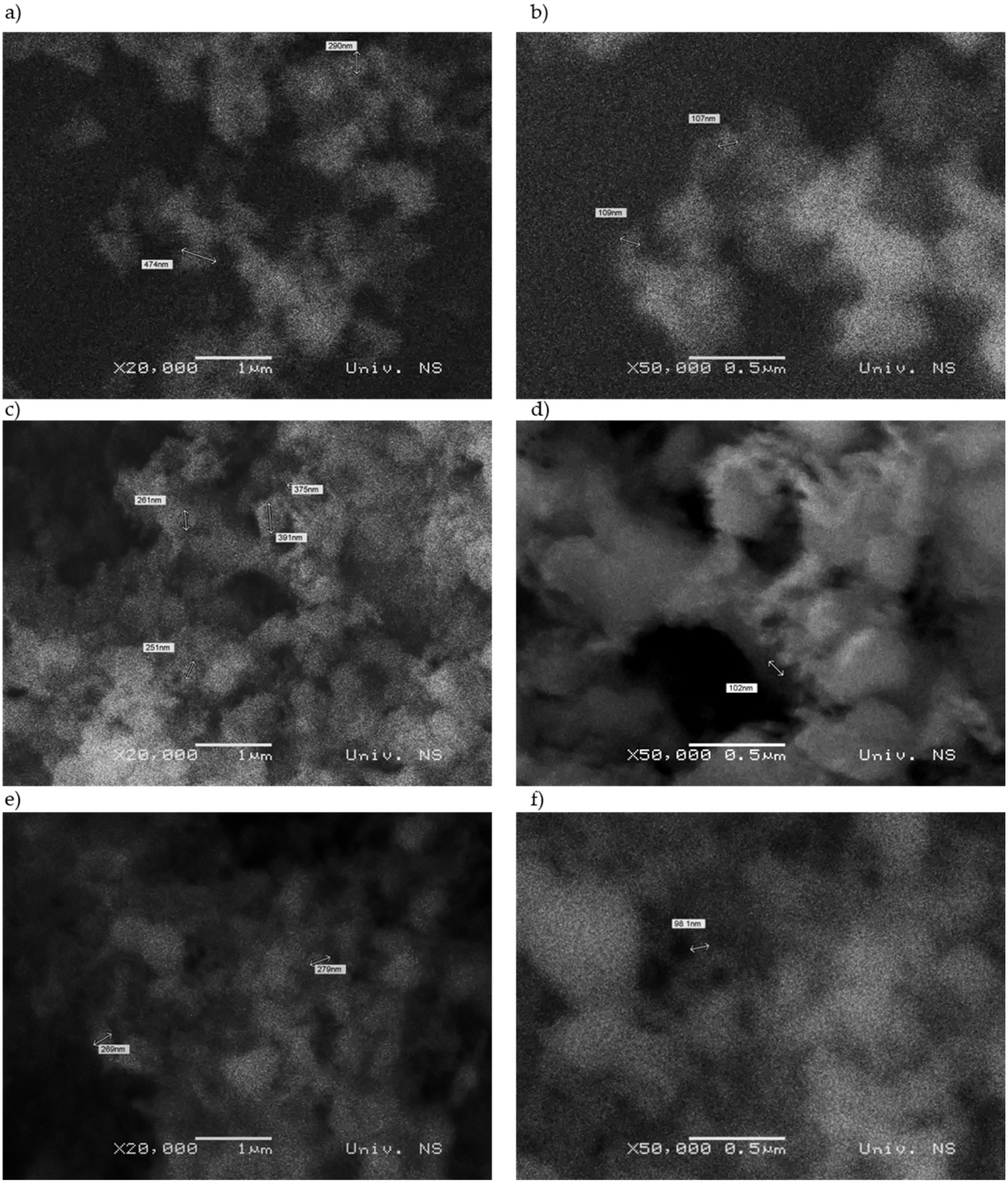

Figure S1. SEM images at different magnification of a), b) green tea; c), d) hibiscus, e) f) lemon balm dust ash

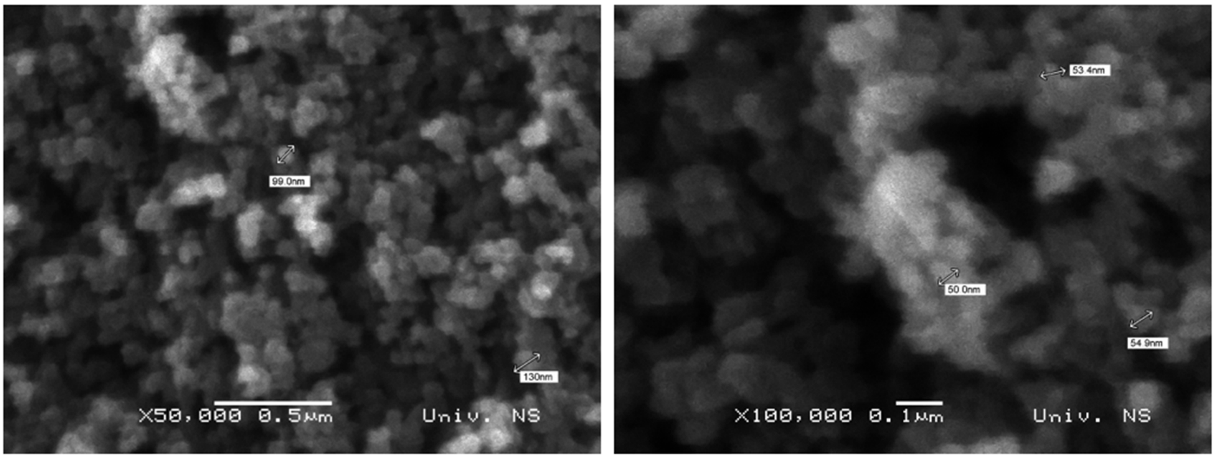

Figure S2. SEM images of carbon black

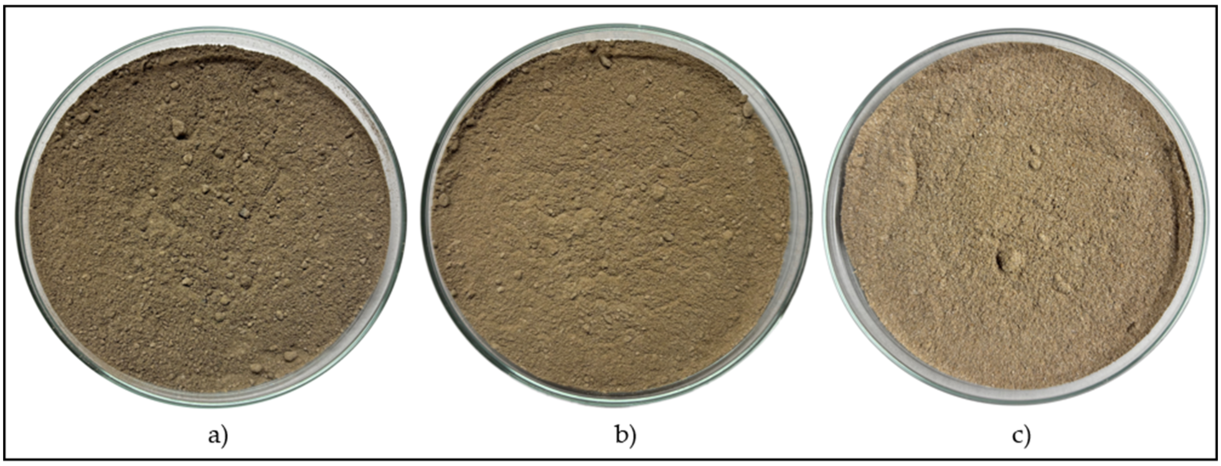

Figure S3. Herbal dust ash after two-step annealing: a) green tea, b) hibiscus, c) lemon balm
